# The effect of multimodal rehabilitation program on pain, functional outcomes, and plantar fascia thickness in patients with plantar fasciitis: a randomized controlled trial

**DOI:** 10.1186/s12891-026-09562-x

**Published:** 2026-02-20

**Authors:** Mohamed Ramadan Ibraheem, Mohamed Ashraf Abd El-Moneim, Mona Mohamed Ibrahim

**Affiliations:** 1 Department of Physical Therapy for Musculoskeletal Disorders and its Surgery, Faculty of Physical Therapy, Cairo University, Giza, Egypt; 2 Department of Physical Therapy for Orthopedic Surgery, Faculty of Physical Therapy, Horus University - Egypt, New Damietta, Egypt; 3 Department of Radiodiagnosis, Faculty of Medicine, Al-Azhar University, New Damietta, Egypt

**Keywords:** Plantar fasciitis, Heel pain, Fascia thickness, Hyperpronation, Foot posture index, Rehabilitation, Multimodal

## Abstract

**Background:**

Plantar fasciitis (PF) is characterized by heel pain and functional limitations, with hyperpronation identified as a primary risk factor. Mechanical correction of malalignment is one of several therapeutic options, but no one has settled on a single, comprehensive treatment protocol. The aim of this study was to examine the effect of a multimodal rehabilitation program that incorporates both active and passive correction of the foot’s pathomechanics on pain intensity, function, plantar fascia thickness, foot posture, and ankle dorsiflexion range of motion in patients with PF, in comparison to a conventional rehabilitation protocol.

**Methods:**

A randomized controlled trial involving 48 patients (21 males and 27 females) aged 40 to 60 years and clinically diagnosed with PF. They were randomly assigned to either the experimental or control groups. The control group received a conventional rehabilitation program (self-stretching, mobilization of the ankle and subtalar joint, myofascial release, and ultrasound), while the experimental group received the multimodal rehabilitation program that included low-dye tape, short foot exercise, and unilateral heel raising, in addition to the same self-stretching and mobilization given to the control group. Both groups were given 3 sessions per week for 8 consecutive weeks (2 months). Outcome measures included pain intensity (using visual analogue scale and pressure pain threshold) and foot posture (via the foot posture index and rear foot angle. Functional impairment (using the foot function index). Dorsiflexion range of motion (assessed with a bubble inclinometer), and plantar fascia thickness (measured via ultrasonography).

**Results:**

Post-treatment, both groups showed significant improvements in all outcomes (*p* < 0.001), except for the foot posture index, which wasn’t significant in the control group (*p* = 0.097). However, the rear foot angle and foot posture index showed significant differences between groups, favoring the experimental group (*p* = 0.046 and *p* = 0.027, respectively).

**Conclusion:**

Both programs effectively improved clinical outcomes and reduced plantar fascia thickness in patients with PF. However, the multimodal rehabilitation program demonstrated greater improvement in foot posture measures. Thorough rehabilitation targeting clinical outcomes and biomechanical aspects may yield superior results in the management of PF.

**Trial registration:**

The trial was registered on June 8, 2024, on clinicaltrials.gov (NCT06456944). https://clinicaltrials.gov/study/NCT06456944.

## Background

Plantar fasciitis (PF) is the most common musculoskeletal disorder causing heel pain; it affects individuals of all ages and activity levels [1]. Plantar fasciitis is usually present unilaterally but may occur bilaterally in 30% of cases [2]. PF accounts for 15% of foot problems in the general population and has a lifetime incidence of around 10%, primarily affecting those aged 40 to 60 years old [3]. Among athletes, runners have a higher incidence, up to 17.4% [4].

Histologically, PF isn’t an accurate term; it involves degenerative changes and minor tears in the plantar fascia insertion into the calcaneus, rather than inflammation, making terms like fasciosis or fasciopathy more precise descriptors [5].

Common symptoms of PF include pain at the medial calcaneal tubercle, particularly noticeable in the first morning steps or when standing after prolonged sitting, and during prolonged weight-bearing activities [6]. These symptoms can lead to functional impairments and decreased quality of life. Risk factors for PF include prolonged standing [7], inappropriate footwear, limited ankle dorsiflexion and big toe mobility [8], foot hyper-pronation [9], tight Achilles tendon, and high body mass index [10].

The exact pathology of PF remains unclear but is believed to have a biomechanical origin. The plantar fascia’s primary role is to stabilize the medial longitudinal arch (MLA) of the foot, which is essential for creating a rigid lever during the propulsive phase of gait through the windlass mechanism [11].

A common risk factor for PF is hyperpronation [9], which involves adduction and plantar flexion of the talus during weight bearing, causing calcaneal eversion, flattening of the MLA, and increased tension in the plantar fascia [11]. This results in microtears and chronic degeneration of the plantar fascia, along with weakness in the intrinsic and extrinsic foot muscles that support the MLA [5].

Additionally, plantar fascia thickening and pain intensity were positively correlated with midfoot loading and foot pronation in PF patients [12]. Therapeutic interventions used in treating PF vary in effectiveness according to the level of evidence. Stretching of the plantar fascia and calf muscles, manual treatments, tapping, and night splints have level A of evidence, while strengthening exercises, foot orthotics, lasers, and dry needling have level B of evidence [6].

According to a recent systematic review, individuals with PF experience short-term benefits from mechanical treatments designed to reduce mechanical loading and stress placed on the plantar fascia [13].

Stretching of the plantar fascia and calf muscle alleviates pain and improves function [14]. High-load resistance training shows quicker pain relief and superior functional improvements compared to stretching alone [15]. Manual therapy techniques, including joint and soft tissue mobilization combined with stretching or strengthening, also effectively reduce pain and improve function [16]. Low-dye taping is beneficial for short-term pain reduction in combination with other treatments for PF [17].

The multimodal rehabilitation program utilized in this study focused on both active and passive correction of the pathomechanics of the foot. Passive correction involved low-dye taping and ankle joint mobilization to improve ankle mobility, along with stretching of the plantar fascia and Achilles tendon, while the active correction involved strengthening of the intrinsic foot muscles and unilateral heel-raising exercise targeting the windlass mechanism.

The aim of this study is to examine the effect of a multimodal rehabilitation program on pain intensity, function, plantar fascia thickness, foot posture, and ankle dorsiflexion range of motion (ROM) in patients with PF and to compare this effect with that of the conventional rehabilitation protocol.

## Methods

### Study design

This study was a prospective, single-blinded, randomized controlled trial. It was conducted at the outpatient clinic of the Faculty of Physical Therapy, Horus University - Egypt, from June 2024 to April 2025. The study was registered at clinicaltrials.gov with the identifier (NCT06456944) and also received approval from the research ethical committee at the Faculty of Physical Therapy, Cairo University (P.T.REC/005275). This study followed the international ethical guidelines developed and reported in the Declaration of Helsinki.

This study was done in consistent with the CONSORT 2025 guidelines for reporting randomized controlled trials.

### Participants

Sample size determination was performed using the G*power software version 3.1.9.7 (Heinrich-Heine-University, Düsseldorf, Germany). The calculation was based on *p* = 0.05, power = 80%, allocation ratio 1:1, and MANOVA repeated measures, within-between interaction using a moderate effect size of 0.502 [18]. The sample size consisted of 48 participants. An additional 16% (8 participants) was added to account for any dropouts or losses in follow-up during the study period in each group. Each group consisted of 28 participants.

Patients were included if they met the inclusion criteria based on the clinical diagnostic guidelines from the American Physical Therapy Association (APTA) [6]: experiencing unilateral heel pain for more than 3 months, most noticeable with the first morning steps or following a period of rest, and potentially worsening with prolonged weight-bearing (those with bilateral PF; only the more symptomatic foot was included in the study; the other foot received advice about performing self-stretching and ice application at home, and its data were not taken). Participants demonstrated tenderness upon palpation of the medial calcaneal tubercle, yielded positive results on the windlass test, exhibited foot pronation as per the foot posture index [6], and were aged between 40 and 60 years, including both genders [1]. The exclusion criteria included a body mass index (BMI) > 30 kg/cm^2^, a history of lower limb injury, such as fractures, surgeries, or malignancies, tarsal tunnel syndrome, atrophy of the fat pad, or corticosteroid treatment within the preceding six months [18].

### Randomization

Patients were assigned to a control group (A) or an experimental group (B) through simple randomization. This allocation process was concealed using sealed, opaque, sequentially numbered envelopes. A total of 56 envelopes were prepared, with 28 participants in the control group (A) and 28 in the experimental group (B).

Patients were blinded to their group assignment. A research assistant, who was blinded to the group assignments, opened the envelopes to reveal each participant’s group allocation, which was indicated by the A or B letter on the envelope.

Each participant received a thorough description of the study’s aims and procedures (to compare the effectiveness of two different programs and determine which is more effective in reducing pain, improving function, and other outcomes; the duration of the treatment; and the pre- and post-measurements) along with possible risks before they were included in the study. All participants signed an informed consent form for their approval to participate in the current study.

Demographic data, including age, weight, body mass index (BMI), gender, and the affected side, were collected pre-treatment. While, at baseline and after 2 months of treatment, patients were assessed for pain intensity by visual analogue scale (VAS) (primary outcome), plantar fascia thickness using ultrasonography, foot posture by foot posture index (FPI), rear foot angle (RFA) by Kinovea software, dorsiflexion range of motion (ROM) using bubble inclinometer, foot function using foot function index (FFI), and pressure pain threshold (PPT) using manual Pressure algometer (secondary outcomes). All outcome measures were assessed by an R.M. (first author), who was trained in all assessment procedures.

### Assessment procedures

#### Pain intensity

The Visual Analog Scale (VAS) is a valid tool for measuring pain intensity, utilizing a 10-centimeter horizontal line. Scores range from 0 (no pain) to 10 (worst imaginable pain), with the patient marking a point that represents their pain level at the first morning steps, which was the primary outcome. The distance in millimeters from the “no pain” end to the mark indicates the pain score, expressed from 0 to 100 mm [19]. It was reported to have a minimal clinically important difference (MCID) of 19 mm on the 100 mm VAS for first morning steps pain [20].

#### Plantar fascia thickness

**Fig. 1:**
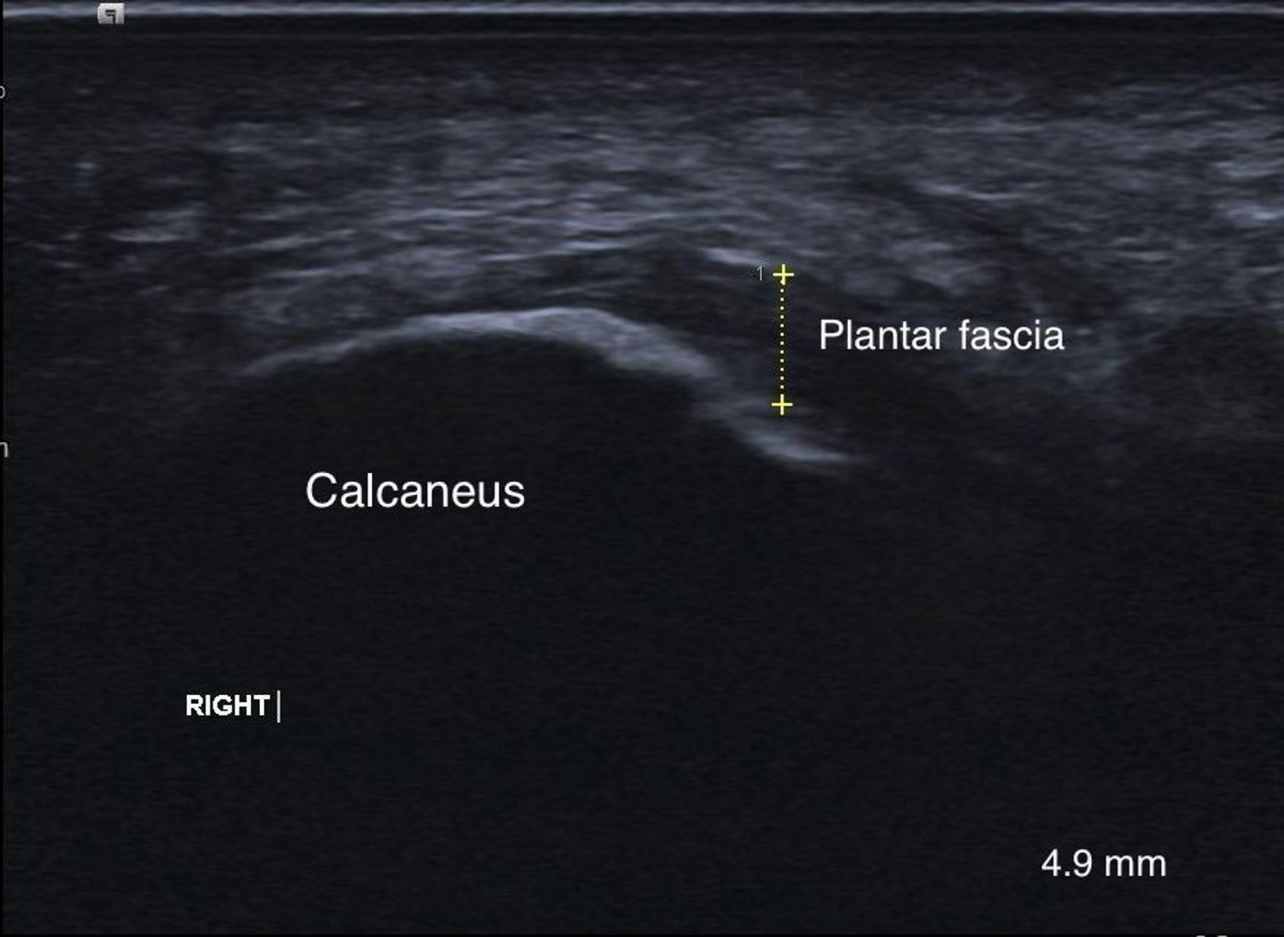
Measurement of plantar fascia thickness

Ultrasonography (US) is a reliable, non-invasive imaging technique used to assess plantar fascia thickness and echogenicity, monitor treatment effectiveness, and guide therapeutic procedures for PF [21]. Measurements are taken with a 10–15 MHz linear array transducer (Acuson Juniper diagnostic ultrasound system; Siemens Medical Solutions USA, Inc., Issaquah, WA, USA). Patients are positioned prone with their ankle in a neutral position, and toes extended to stretch the plantar surface and clarify the fascia’s borders. Measurement of plantar fascia thickness was done by A.M. (third author), an experienced radiologist with 14 years of experience. He performed a longitudinal-axis US approximately half a centimeter medial to the midpoint of the plantar surface, targeting the fascia’s insertion on the calcaneus as represented in Fig. 1. The average of three consecutive scans was recorded [22].

#### Foot posture

The six-item Foot Posture Index (FPI-6), introduced by Redmond et al. in 2006, is a quick, reliable clinical tool for assessing foot posture [23]. It demonstrates excellent inter-rater reliability [24] and has been used to recruit patients with foot pronation. The FPI-6 comprises six factors evaluated while standing: talus palpation, malleolus curvature, calcaneus position, talonavicular prominence, medial longitudinal arch congruence, and forefoot-rearfoot alignment. Each factor is scored from − 2 (supination) to + 2 (pronation), with 0 representing a neutral position. The summed total score ranges from − 12 to + 12, with higher positive scores indicating a more pronated foot [23].

#### Rear foot angle

Kinovea is a free 2D motion analysis software. It has demonstrated validity and reliability (both inter- and intra-rater) for measuring angles and distances up to 5 m, with optimal results at a 90-degree angle [25]. Kinovea version 2023.1.2 was used to measure RFA. An Apple iPhone 13 with a dual 12-megapixel camera system (Apple Inc., Cupertino, California, USA) was used to capture back-view photos. Patients were asked to stand on the ground, and the camera was placed on a tripod perpendicular to the ground, adjusted to ankle level, about 1.5 m away from the patient. Three markers were placed at the base of the calcaneus, the middle of the gastrocnemius, and the posterior aspect of the talus. A back-view photo was taken by a camera for each patient, and the image was subsequently exported to Kinovea to measure RFA. Rear foot angle was calculated as the angle between the line extending from the posterior aspect of the talus to the middle of the gastrocnemius muscle and the calcaneus. If the angle was negative, it means eversion, and if it was positive, it means inversion [26].

#### Dorsiflexion range of motion

The bubble inclinometer showed high reliability in measuring weight-bearing ankle dorsiflexion ROM, with interclass correlation coefficients (ICCs) ranging from 0.96 to 0.99 and a low standard error of measurement (SEM) when compared to a goniometer. The MCID for the inclinometer ranged from 3.7° to 3.8° [27]. A Baseline Bubble Inclinometer, model 10,602 (Fabrication Enterprises Inc., New York, USA), was used to measure ankle dorsiflexion ROM during a weight-bearing lunge against a wall [28]. Participants were instructed to lunge maximally toward their toes, keeping their heel on the ground and their knee aligned with the second toe. The inclinometer was secured to the lateral side of the distal leg, just above the ankle joint, with a Velcro strap, oriented laterally, with its zero-point parallel to the floor [29].

#### Foot function

The Arabic version of the Foot Function Index (FFI) demonstrates good validity and reliability for assessing pain, disability, and activity limitations in Arabic-speaking patients with foot and ankle disorders [30]. The FFI comprises 23 items across three subscales: pain (9 items), activity limitation (5 items), and disability (9 items) [31]. For patients with PF, the MCID for the FFI pain subscale is 12 points, and for the disability subscale, it is 7 points [32]. Scoring involves marking items on a VAS from “No pain” to “Worst pain possible,” with scores ranging from 0 to 9 for each question. “Not applicable” (NA) responses exclude questions from scoring. Subscale scores are calculated by dividing the total score by the maximum potential score of applicable questions, then multiplying by 100 to yield a 0-100 range, where higher scores indicate greater disability. The total FFI score is the mean of these three subscale values [31].

#### Pressure pain threshold

The pain pressure threshold (PPT), defined as the minimum pressure causing pain, is an objective indicator of pain intensity and was measured by a manual algometer (Baseline^®^ Pressure Algometer (push-pull force gauge), model 12–0302, Fabrication Enterprises Inc., New York, USA) [33, 34]. It was calibrated in kg/cm^2^. The pressure algometer is valid and reliable to measure PPT and demonstrated a strong correlation when compared with the force plate [35]. The PPT was assessed with the patient in a supine position, knees extended, and feet suspended off a plinth. A therapist applied vertical pressure using a disc at the anteromedial aspect of the heel, the area of maximum tenderness. Pressure was gradually increased at a rate of 0.1 kg/s until the patient reported pain or the pressure itself became painful. Three measurements were recorded with 30-second rest intervals between each, and the mean of these trials was used for analysis [33].

### Treatment procedures

All interventions were administered by R.M. (first author), a licensed physical therapist with 9 years of clinical experience. Due to the nature of the treatment, the therapist was not blinded to group allocation. Patients in the control group received a conventional rehabilitation program, including self-stretching, mobilization, plantar fascia release, and ultrasound, while patients in the experimental group received the multimodal rehabilitation program, including low-dye taping, short foot exercise, and high-load strength training combined with the same self-stretching and mobilization applied in the control group. Both groups received 3 sessions per week for 2 months.

The self-stretching regimen involved plantar fascia-specific stretching (PFST) and calf muscle stretching. The PFST was performed while the patient was seated, with the affected foot placed on the thigh of the unaffected leg. Patients grasped the base of the toes and passively extended them until a stretch of the plantar fascia was felt. They were instructed to start slowly and gradually increase the stretch as tolerated [36].

The calf muscle stretching involved stretching of the gastrocnemius and soleus muscles. The patient was asked to stand with the affected leg behind the non-affected leg and lean forward without heel raising until he felt a stretch in the Achilles tendon or the muscle itself. To stretch the gastrocnemius, the affected leg was kept straight, and to stretch the soleus, it was flexed. The stretches were done twice daily, with three sets of three repetitions with a 30-second hold for each muscle [37].

Patients in both groups received mobilization of the talocrural joint, mobilization with movement to improve dorsiflexion range of motion (ROM), and mobilization of the subtalar joint to enhance inversion and eversion ROM. Each technique was done for 1.5 min [38].

**Fig. 2:**
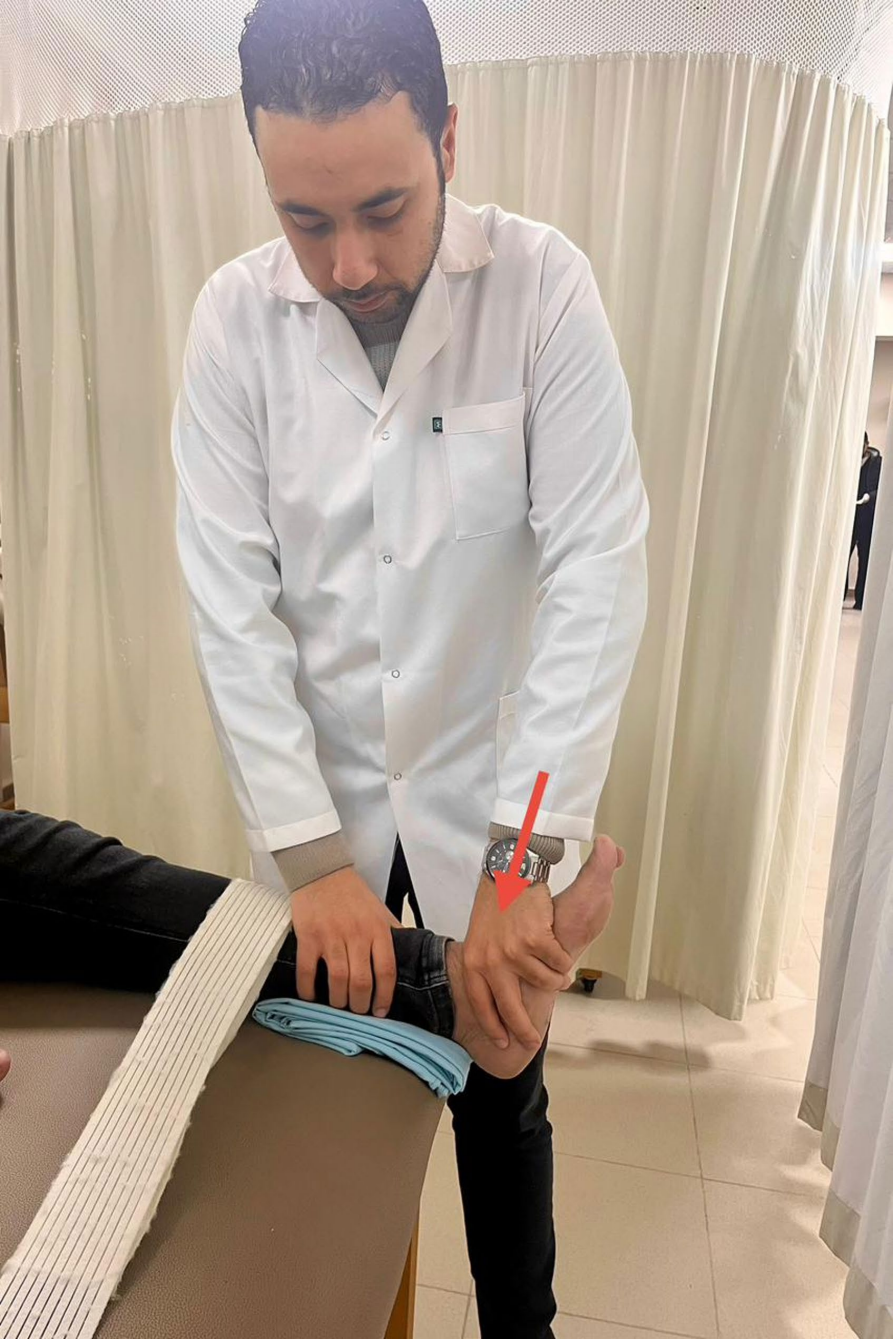
Posterior glide of the talus

**Fig. 3:**
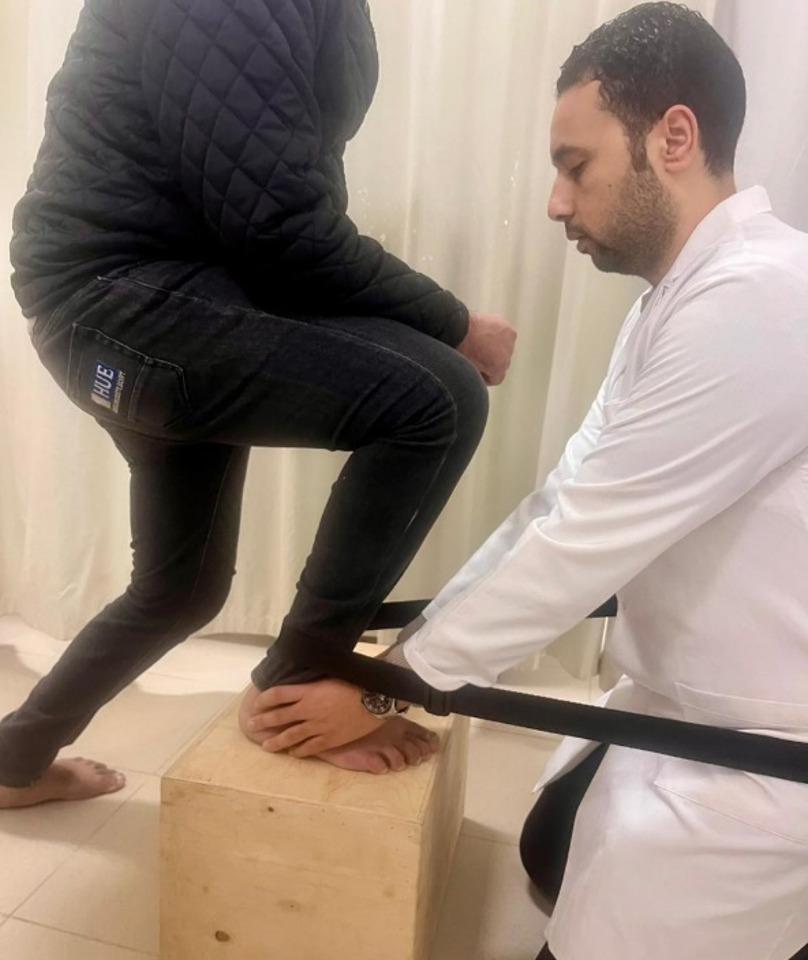
Mobilization with movement to increase dorsiflexion

Mobilization of the talocrural joint involved patients lying supine with their legs supported by a belt. The therapist supported the distal tibia and fibula with one hand while the other hand applied a grade one caudal distraction and posterior talar glide [38] (Fig. 2). During mobilization with movement (MWM), patients stood on a chair with their feet facing the therapist, who then performed a posterior talar glide. The patients were asked to lunge forward, assisted by a padded belt placed between the therapist’s waist and the distal lower leg of the patient, while maintaining the posterior glide of the talus [39] (Fig. 3).

**Fig. 4:**
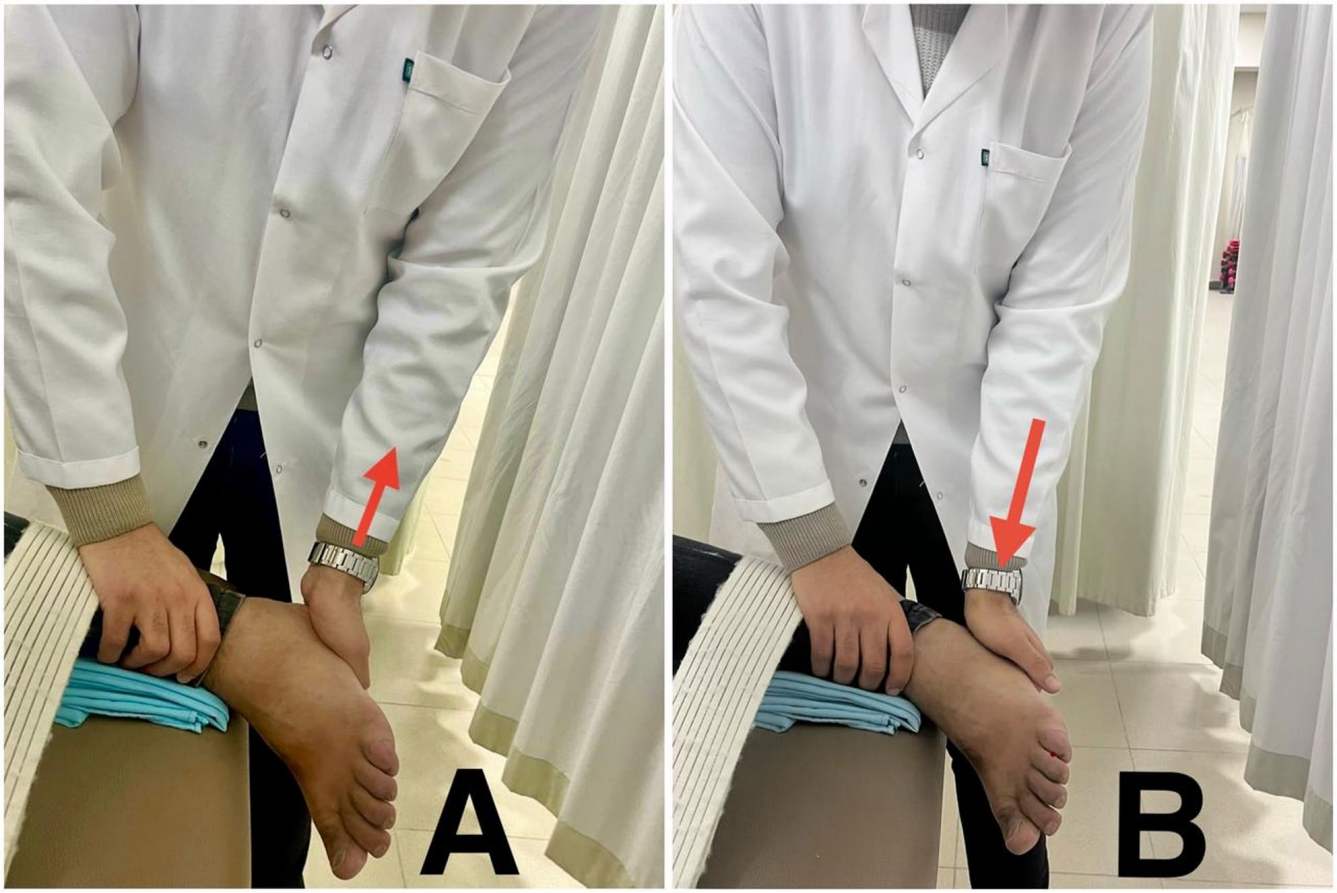
**A**: lateral glide of the subtalar joint. **B**: medial glide of the subtalar joint

Mobilization of the subtalar joint was done while patients were in a side-lying position. The therapist stabilized the talus with one hand, and the other hand grasped the calcaneus. Lateral glide was done to improve eversion (Fig. 4A), and medial glide was done to improve eversion [38] (Fig. 4B).

Plantar fascia release was performed with the patient positioned prone, the knee extended, the calcaneus everted, and the talocrural joint dorsiflexed. The therapist carried out toe extension and proximal and distal releases of the plantar fascia and flexor hallucis longus for 5 min each session using the thumb. The extent of the release was guided and progressed by the patient’s reactions and tolerance [39].

Ultrasound therapy using a PhysioGo 200 A ASTAR (Bielsko-Biała, Poland) was administered to patients in a prone position. The treatment involved a pulsed mode with a 50% duty cycle, 1.5 W/cm^2^ intensity, and a 1 MHz frequency for deeper penetration. Applied for 5 min to the most tender point on the proximal insertion of the plantar fascia, a thin layer of gel was used between the ultrasound head and skin for optimal transmission [37].

**Fig. 5:**
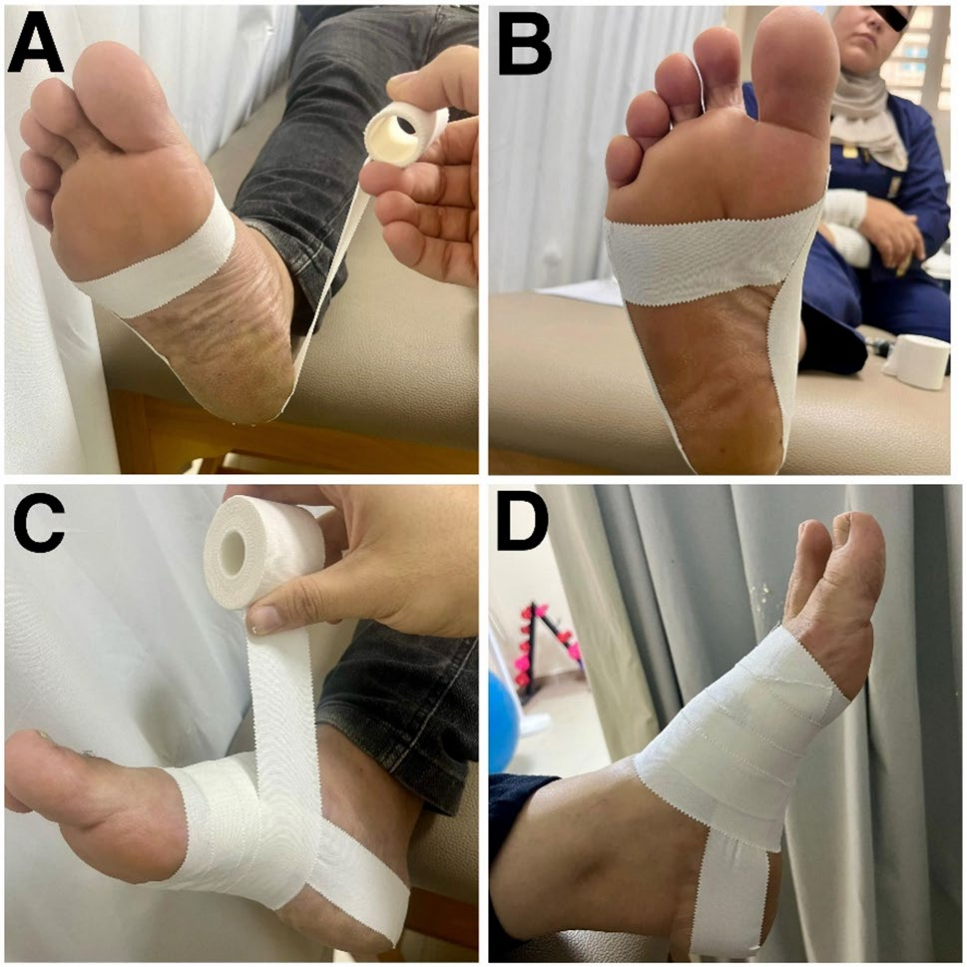
Step-by-step application of the low-dye taping technique.** A:** An anchor strip was applied without tension around the metatarsal heads. **B:** The first corrective "U" strip was applied with tension from the lateral to the medial aspect of the foot to support the medial longitudinal arch. **C:** Multiple overlapping strips were added to reinforce the support of the arch. **D:** The final appearance of the tape

Low-Dye taping was performed using 5 cm wide rigid tape (Falcon^®^, Sports Medical, China). Before applying low-dye taping, participants washed and dried their feet. The first tape strip was applied without tension around the forefoot near the metatarsal heads to anchor the subsequent strips (Fig. 5A). The second strip, applied proximal to the fifth metatarsal head, wrapped posteriorly around the calcaneus to the first metatarsal head, with tension on the medial side to invert the hindfoot and adduct the forefoot (Fig. 5B). Three to four additional strips were placed similarly, overlapping the previous strips by half their width, with the final strip positioned distal to the talocrural joint (Fig. 5C). Tension on these strips supported the MLA by pulling on the medial surface of the midfoot. The final appearance of the tape is represented in (Fig. 5D). Taping was maintained for 24 h [40].

Short foot exercises (SFE) are more effective than towel curling for activating the abductor hallucis muscle and maintaining the medial longitudinal arch (MLA). The exercise involves standing with feet shoulder-width apart, then actively supinating the foot to raise the MLA by bringing the first metatarsal head toward the heel, without toe flexion. Patients are told to stand on one leg with a slightly bent knee for 10 s while holding the MLA height. Then, over the course of 5 s, they should slowly lower the MLA with eccentric control. This concentric, isometric, and slow eccentric SFE can be performed in up to five sets, multiple times daily. Progression includes unsupported single-limb support with eyes open and then closed [41].

**Fig. 6:**
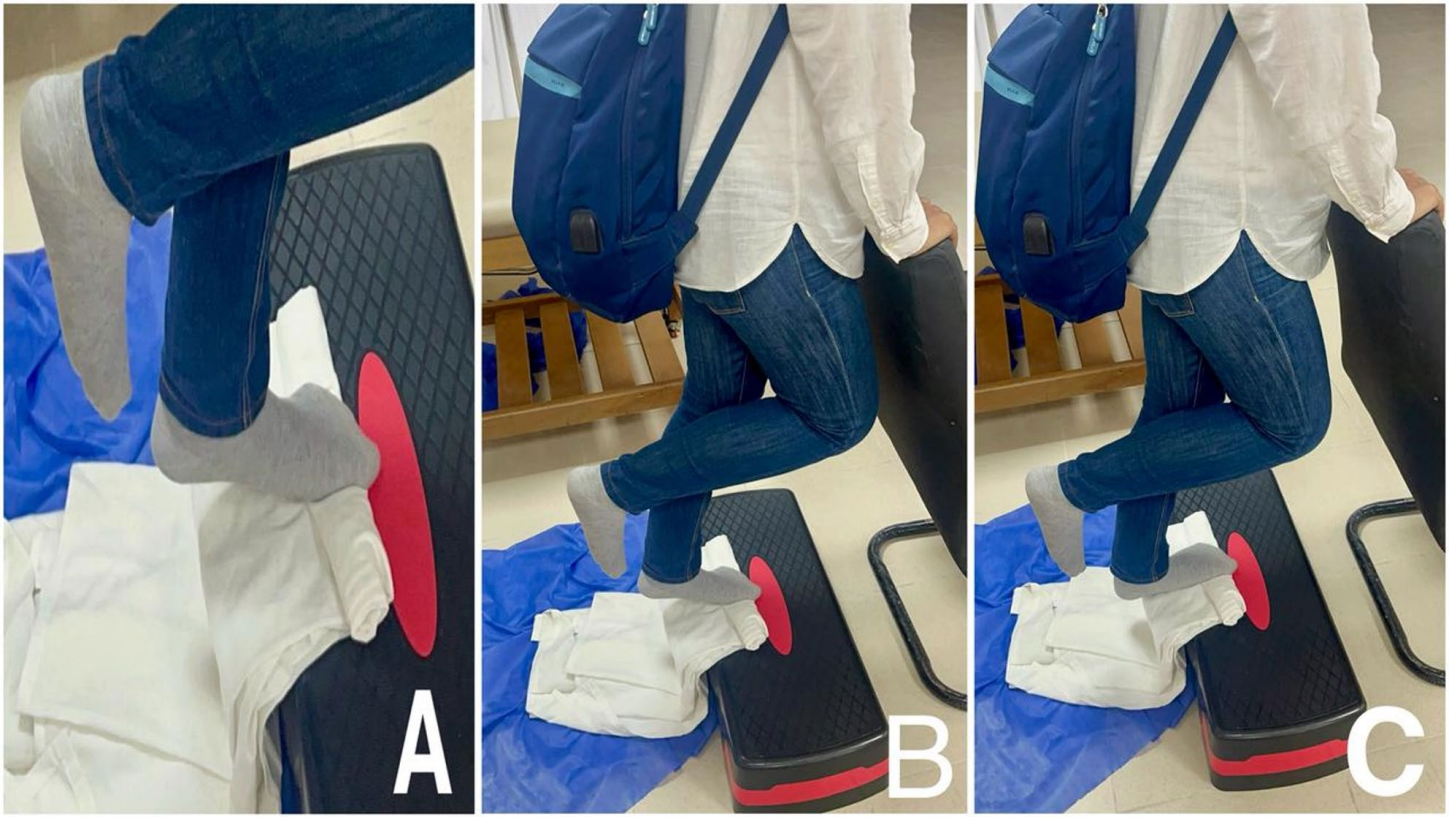
Unilateral heel raise exercise.** A:** The starting position for the exercise, with the patient standing on a step and their feet dorsiflexed over a towel to stimulate the windlass mechanism. **B:** The concentric phase of the exercise. **C:** The eccentric phase of the exercise

The unilateral heel raise exercise, designed to activate the windlass mechanism, involves standing on a step with toes fully dorsiflexed, achieved by placing a towel under them (Fig. 6A). The exercise consists of a 3-second concentric phase (Fig. 6B), a 3-second eccentric phase (Fig. 6C), and a 2-second isometric hold. Patients performed this exercise three times weekly for two months. Progression began with three sets of 12 repetitions at maximum (RM), with the weight increased using a backpack of books after two weeks. Individuals who were unable to complete unilateral raises initially started with bilateral raises and transitioned to unilateral raises as their strength increased. The exercise progression followed a protocol: Weeks 1–2 involved 3 sets of 12 RM; weeks 3–4 progressed to 4 sets of 10 RM; and weeks 5–8 progressed to 5 sets of 8 RM according to the protocol outlined by Rathleff et al. [15].

### Data analysis

Patients’ demographic characteristics were compared between groups utilizing independent t-tests. The chi-squared test was used to compare the gender and affected side among groups. To check if the data followed a normal distribution, the Shapiro-Wilk test was used. The homogeneity of variance among groups was checked utilizing Levene’s test. A mixed-design 2 × 2 MANOVA test has been employed to compare the effects of time (pre- and post-) and treatment (between groups) on all dependent variables, as well as the interaction between these two factors. For the subsequent multiple comparisons, post hoc tests with the Bonferroni correction were conducted. The level of significance for all statistical tests was set at *p* < 0.05. SPSS version 28 for Windows (*IBM SPSS*,* Chicago*,* IL*,* USA*) was used to conduct all of the statistical analyses.

## Results

**Fig. 7:**
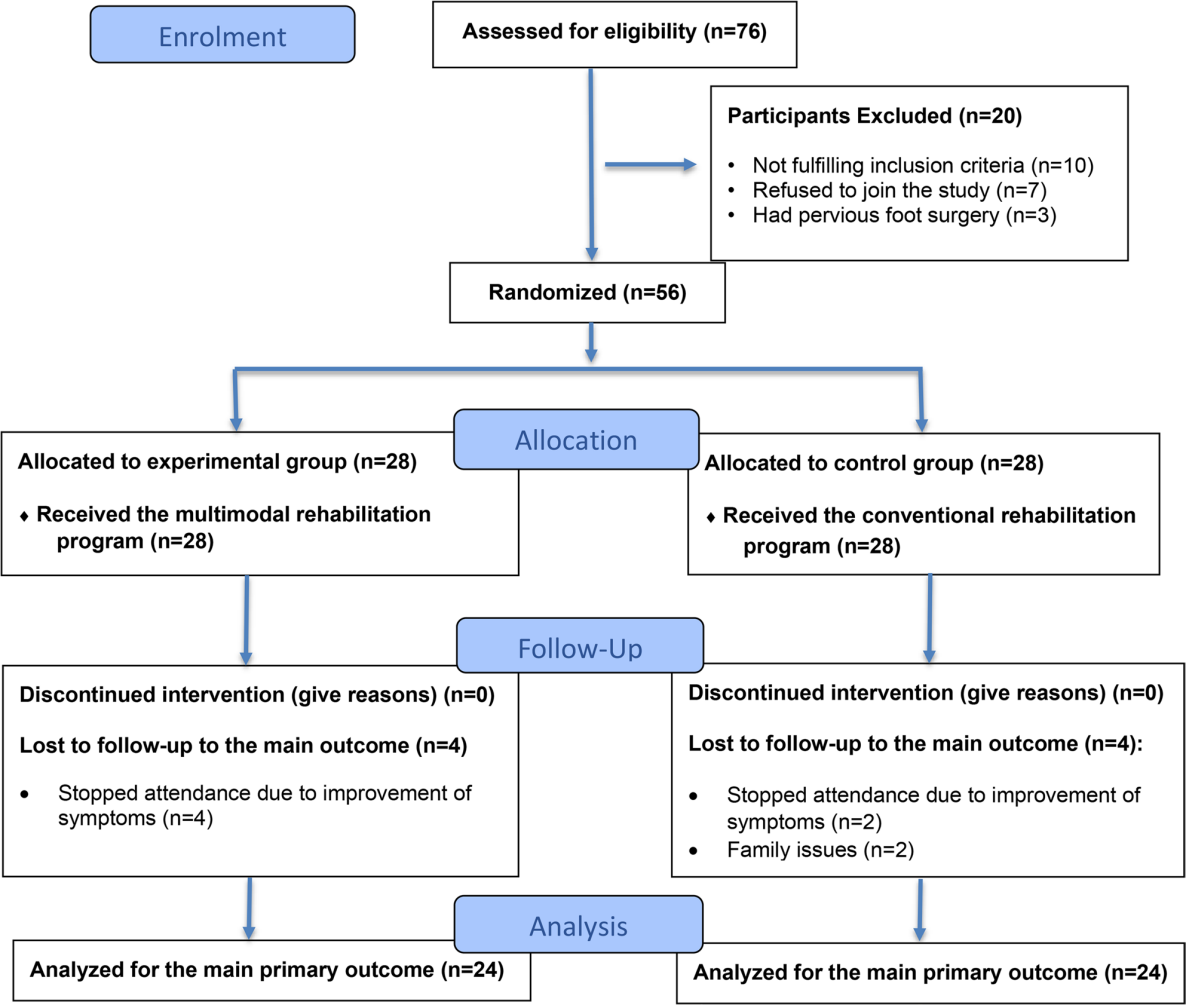
Patients flow chart

Seventy-six patients with PF were screened for inclusion in this study. Ten did not meet the inclusion criteria, and seven refused to participate, while three had previous foot surgery. Fifty-six patients were randomly assigned to be 28 in the experimental group and 28 in the control group. At follow-up, 4 patients in the experimental group and 2 in the control group stopped attending due to symptom improvement, and an additional 2 in the control group were lost to follow-up due to family issues. Forty-eight patients (21 men and 27 women) completed the study, as shown in Fig. 7.

### Patients’ general characteristics

Patients in both groups showed similar baseline characteristics, with no significant differences observed between them (*p* > 0.05), as shown in Table 1.

**Table 1:** Patients’ general characteristics in both groups

Variables	Groups (Mean ± SD)		*p*-value
	Control group (*n* = 24)	Experimental group (*n* = 24)	
Age (years)	50.37 ± 6.26	47.70 ± 4.75	0.103
Weight (kg)	88.91 ± 19.29	85.41 ± 8.28	0.418
Height (cm)	168.25 ± 18.77	173.25 ± 7.55	0.232
BMI (kg/m ^2^ )	28.74 ± 1.74	28.37 ± 1.42	0.413
Gender (Males: Females)	10 (41.7%): 14 (58.3%)	11 (45.83%): 13 (54.17%)	0.771
Affected side (Right: Left)	13 (54.2%): 11 (45.8%)	13 (54.2%): 11 (45.8%)	0.99

Quantitative data (age, weight, height) are reported as mean ±standard deviation and compared by a t-independenttest

Qualitative data (gender, affected side) are reported as frequency (percentage) and compared by Chi-square test

*p*-value: probability value; *p*-value > 0.05: non-significant

### Analysis and interpretation of results of both groups pre- and post-treatment

Both groups demonstrated a highly significant improvement in pain measured by VAS scores and PPT (*p* < 0.001). However, no significant differences were found between groups in VAS and PPT (*p* = 0.764) and (*p* = 0.801), respectively, as shown in Table 2.

Both groups demonstrated a highly significant improvement in FFF (pain, disability, activity limitations, and total score) (*p* < 0.001). However, no significant differences were found between groups (*p* = 0.436), (*p* = 0.274), (*p* = 0.460), and (*p* = 0.330) respectively, as shown in Table 2.

Both groups demonstrated a highly significant improvement in dorsiflexion ROM and thickness (*p* < 0.001). However, no significant differences were found between groups (*p* = 0.722) and (*p* = 1.000) respectively, as shown in Table 2.

Both groups demonstrated a highly significant improvement in RFA (*p* < 0.001); however, there was a statistically significant improvement in RFA favoring the experimental group (*P* = 0.046), with a percent of change of 35.86% in the experimental group, while it was 15.94% in the control group, as shown in Table 2.

No significant within-group improvement in FPI in the control group (*p* = 0.097). However, there was significant improvement within and between groups in FPI, favoring the experimental group (*P* = 0.027) with a percentage of change of 43.06%, while it was 7.5% in the control group, as shown in Table 2. 

**Table 2:** Within and between groups comparison for all measured variables:

Variables	Items	Groups (Mean ± SD)		Between group MD (95% CI)	F-value	*P*-value
		Control group (*n* = 24)	Experimental group (*n* = 24)			
VAS (Scores)	Pre-treatment	78.42 ± 14.44	79.50 ± 12.25	-1.08 (-8.86; 6.7)	0.079	0.781
	Post-treatment	11.62 ± 8.27	12.29 ± 5.68	-0.67 (-4.79; 3.46)	0.106	0.746
	Within group (MD) (95% CI)	66.79 (61.41; 72.18)	67.21 (61.82; 72.59)			
	Change %	85.18%	84.54%			
	*P*-value	**< 0.001** *	**< 0.001** *			
Foot function index (Pain) (Scores)	Pre-treatment	80.29 ± 12.13	83.60 ± 10.08	-3.31 (-9.79; 3.17)	1.059	0.309
	Post-treatment	14.74 ± 10.81	17.07 ± 9.66	-2.33 (-8.29; 3.63)	0.618	0.436
	Within group (MD) (95% CI)	65.55 (59.2; 71.9)	66.53 (60.18; 72.88)			
	Change %	81.63%	79.58%			
	*P*-value	**< 0.001** *	**< 0.001** *			
Foot function index (Disability) (Scores)	Pre-treatment	74.22 ± 14.31	76.02 ± 13.23	-1.795 (-9.80; 6.21)	0.204	0.654
	Post-treatment	9.56 ± 7.81	11.97 ± 7.32	-2.42 (-6.82; 1.98)	1.223	0.274
	Within group (MD) (95% CI)	64.67 (58.47; 70.86)	64.04 (57.85; 70.24)			
	Change %	87.13%	84.25%			
	*P*-value	**< 0.001** *	**< 0.001** *			
Foot function index (Activity limitation) (Scores)	Pre-treatment	59.42 ± 19.84	64.82 ± 16.37	-5.40 (-15.96; 5.17)	1.056	0.309
	Post-treatment	6.01 ± 9.12	8.02 ± 9.49	-2.00 (-7.41; 3.40)	0.556	0.460
	Within group (MD) (95% CI)	53.41 (45.26; 61.56)	56.80 (48.65; 64.96)			
	Change %	89.88%	87.64%			
	*P*-value	**< 0.001** *	**< 0.001** *			
Foot function index (Total) (Scores)	Pre-treatment	74.13 ± 12.94	77.04 ± 11.07	-2.92 (-9.91; 4.08)	0.703	0.406
	Post-treatment	10.92 ± 8.44	13.22 ± 7.73	-2.30 (-7.00; 2.41)	0.967	0.330
	Within group (MD) (95% CI)	63.20 (57.29; 69.12)	63.82 (57.90; 69.74)			
	Change %	85.27%	82.84%			
	*P*-value	**< 0.001** *	**< 0.001** *			
Foot posture index (Scores)	Pre-treatment	4.00 ± 1.67	4.83 ± 2.10	-0.83 (-1.94; 0.27)	2.319	0.135
	Post-treatment	3.71 ± 1.57	2.75 ± 1.33	0.96 (0.11; 1.80)	5.202	**0.027** *
	Within group (MD) (95% CI)	0.3 (-0.06; 0.64)	2.08 (1.74; 2.43)			
	Change %	7.5%	43.06%			
	*P*-value	0.097	**< 0.001** *			
Plantar fascia thickness (mm)	Pre-treatment	5.79 ± 1.12	5.51 ± 0.89	0.28 (-0.31; 0.87)	0.911	0.345
	Post-treatment	4.54 ± 0.71	4.54 ± 0.79	0.0 (-0.44; 0.44)	0.000	0.99
	Within group (MD) (95% CI)	1.25 (0.91; 1.58)	0.97 (0.63; 1.30)			
	Change %	21.45%	17.45%			
	*P*-value	< 0.001 *	**< 0.001** *			
Rear foot angle (degrees)	Pre-treatment	6.21 ± 2.07	6.72 ± 1.27	-0.51 (-1.51; 0.49)	1.048	0.311
	Post-treatment	5.22 ± 1.83	4.32 ± 1.16	0.908 (0.02; 1.80)	4.192	0.046*
	Within group (MD) (95% CI)	0.99 (0.55; 1.42)	2.40 (1.97; 2.84)			
	Change %	15.94%	35.86%			
	*P*-value	**< 0.001** *	**< 0.001** *			
Ankle dorsiflexion ROM (degrees)	Pre-treatment	26.96 ± 4.55	26.33 ± 5.17	0.63 (-2.21; 3.46)	0.197	0.659
	Post-treatment	38.37 ± 4.37	38.00 ± 2.67	0.375 (-1.73; 2.48)	0.129	0.722
	Within group (MD) (95% CI)	-11.42 (-13.4; -9.43)	-11.67 (-13.65; -9.69)			
	Change %	42.37%	44.32%			
	*P*-value	**< 0.001** *	**< 0.001** *			
Pressure pain threshold (kg)	Pre-treatment	1.98 ± 0.55	2.07 ± 0.41	-0.08 (-0.36; 0.20)	0.326	0.571
	Post-treatment	3.65 ± 0.99	3.71 ± 0.53	-0.06 (-0.52; 0.41)	0.064	0.801
	Within group (MD) (95% CI)	-1.66 (-1.97; -1.35)	-1.64 (-1.95; -1.33)			
	Change %	84.34%	79.61%			
	*P*-value	**< 0.001** *	**< 0.001** *			

Data are reported as mean ± standard deviation (SD) and compared statistically by 2 × 2 MANOVA test.

*MD* Mean difference, *CI *confidence interval

*P*-value: probability value

^*^ Significant (*P* < 0.05)

## Discussion

The current study was done to examine the effect of the multimodal rehabilitation program on pain intensity, function, fascia thickness, foot posture, and ankle dorsiflexion ROM in patients with PF, and to compare this effect with that of the conventional rehabilitation protocol.

Both interventions in this study significantly improved clinical outcomes (pain intensity, function, ROM) and plantar fascia thickness post-treatment. However, no statistically significant differences between groups were found, except in FPI and RFA, favoring the experimental group.

In light of that, the suggested protocol aimed to correct foot pathomechanics through both active and passive approaches, incorporating SFE and unilateral heel raises for active correction and low-dye tapping for passive correction; this combination explains the significant improvements in foot posture, measured by FPI and RFA, in the experimental group compared to the control group.

This study utilized low-dye taping, which is superior to other taping techniques [42] in supporting the midfoot and rearfoot by controlling MLA collapse, aligning the calcaneal stance position closer to neutral, increasing lateral midfoot pressure, decreasing forefoot pressure, and reducing calcaneal eversion, all of which contribute to a reduction in foot pronation [43].

The addition of SFE in the experimental group contributed to foot posture improvement through tapping, as it effectively strengthens intrinsic foot muscles that support the MLA. It was reported to enhance foot kinematics, posture, and rear foot alignment, decreasing navicular drop and increasing forefoot inversion, rearfoot adduction, arch height index, and tibial external rotation, linked to elevated MLA height in individuals with foot pronation [44, 45].

However, the role of intrinsic foot muscle strengthening in managing or preventing PF remains inconclusive according to a systematic review [46].

Unilateral heel-raising exercises also contributed to active support of the MLA by exerting high-load forces on the plantar fascia and windlass mechanism, promoting collagen type 1 synthesis. This may help normalize the structures of the tendon and plantar fascia, thereby improving outcomes for patients with degenerative changes in the plantar fascia [15].

A progressive unilateral heel-raising protocol, compared to PFST, demonstrated pain relief and functional improvement in patients with PF over a 3-month program and a 12-month follow-up [15].

The improvement of pain, function, and dorsiflexion ROM may be linked to PFST and calf stretching. Stretching relieves tightness in the plantar fascia and helps it to withstand both regular and excessive stress from loading and activities [47]. Additionally, stretching addresses risk factors for PF, including tight Achilles tendons and restricted ankle dorsiflexion ROM. By enhancing tendon flexibility through stretching, ankle motion is improved, which decreases strain on the fascia and addresses one of the mechanical deficits related to PF [47].

Taping also provided pain relief and functional improvement by reducing strain on the plantar fascia through supporting the MLA. A cadaver study indicated that a higher arch produces less stress on the plantar fascia than a flattened arch [48]. The pain-relieving effects are attributed to improved proprioceptive feedback or mechanical correction of foot alignment [49].

The effect of joint mobilization enhances the mobility of the calcaneus and talus, reducing traction stress on the plantar fascia, which improves pain, function, and ROM [50].

Both groups in this study utilized treatments like stretching and mobilization, supported by level A clinical guidelines for improving clinical outcomes [6]. These techniques likely contributed to clinical benefits with fewer differences between groups. While the conventional program may be effective enough to produce significant pain and function improvements [37]. Our suggested program resulted in significantly greater improvements in FPI and RFA for the experimental group. However, the follow-up duration, while sufficient for biomechanical changes, may not have been long enough to observe greater clinical outcomes, suggesting that longer follow-up (beyond 2 months) may be necessary for better clinical results compared to the conventional program.

Plantar fascia thickness significantly decreased in both groups but remained above the pain-free range of 2.2–4.0 mm, indicating persistent fascial abnormalities despite reduced symptoms. This supports previous studies indicating that loading-based treatments lead to decreased thickness [15, 51].

The study’s results regarding improvements in pain intensity, function, and ROM are consistent with previous studies on combined treatment methods for patients with PF [39, 52–54].

Cleland et al. supported this study’s findings, demonstrating that a combination of manual therapy techniques, intrinsic foot muscle training, and heel raising significantly enhanced function and alleviated pain in both the short and long term more than manual therapy and electrotherapy alone [39].

Additionally, the results of the study align with Kashif et al., indicating that subtalar MWM, stretching, and rigid tape are more effective than a traditional program in alleviating pain and enhancing functional abilities in patients with PF [52].

Furthermore, Rahane et al. found that adding tapping to traditional treatments (PFST, calf stretching, ultrasound, heel raises, and intrinsic muscle strengthening) was more effective at reducing pain and improving function than traditional treatment alone. The study used kinesiotape instead of rigid tape and involved athletes rather than non-athletic adults [53].

The study’s findings aligned with those of Çil’s study, which evaluated an outpatient program including stretching, strengthening, myofascial release, and mobilization to a home-based stretching and strengthening program for individuals with PF. The outpatient group showed significantly greater improvements in ROM, balance, proprioception, flexibility, and superior FFI and VAS scores during the six-month follow-up period [54].

In contrast to the study’s findings, Kamonseki et al. determined that combining calf and PFST with either intrinsic/extrinsic foot muscle strengthening or foot and hip strengthening was equally effective regarding pain, function, and dynamic balance. This discrepancy might be attributed to the absence of manual therapy and tapping in all three protocols, which could affect the outcomes [14].

This study offers a comprehensive rehabilitation approach for treating PF, focusing on foot hyperpronation correction via a structured program. This approach provides not only biomechanical improvement but also keeps the improvement in pain and functional limitations provided by the conventional treatment, which suggests a more holistic and long-term treatment option.

The current study included some limitations. First, only the short-term effect of the multimodal rehabilitation program was studied. Second, FPI depends on clinician observation, which may cause variability in measurement. This issue was managed by the clinician’s experience and the comparable results with the RFA measurement via Kinovea. Third, neither the assessor nor the therapist was blinded during the evaluation and treatment, increasing the risk of performance bias. This risk was reduced by utilizing patient-reported outcomes (e.g., VAS and FFI) and instrument-based outcomes (e.g., US). Fourth, some participants reported negative effects from using the tape for a long time, such as allergic reactions, skin breakdown, and itching. However, these effects were resolved upon tape removal.

Future studies are needed to examine the long-term effect of the multimodal rehabilitation program, which may detect additional gains in the clinical outcomes.

## Conclusion

Both programs were effective in reducing pain and plantar fascia thickness and improving function and ROM in patients with plantar fasciitis. However, the multimodal rehabilitation program was superior in providing additional improvements in FPI and RFA as measures of foot posture. Therefore, comprehensive rehabilitation strategies targeting both clinical outcomes and underlying biomechanical factors may lead to superior outcomes for the management of PF.

## Data Availability

All the data and materials, including patients’ data sheets, raw data pictures, and videos of practical sessions, are available upon request from the corresponding author.

## Abbreviations

APTA: American Physical Therapy Association
FFI: Foot function index
FPI: Foot posture index
FPI-6: Six-item Foot Posture Index
MCID: Minimal clinically important difference
MLA: Medial longitudinal arch
MWM: Mobilization with movement
PF: Plantar fasciitis
PFST: Plantar fascia specific stretch
PPT: Pain pressure threshold
RFA: Rear foot angle
RM: Repetition maximum
ROM: Range of motion
SFE: Short foot exercise
US: Ultrasonography
VAS: Visual analogue scale
